# Overexpression of LAG-3: a potential indicator of low immune function in tuberculosis

**DOI:** 10.3389/fcimb.2024.1410015

**Published:** 2024-06-18

**Authors:** Yun Pan, Zengxi Yao, Lifen Huang, Meina Xu, Ruichang Chen, Dengsheng Li, Xinyuan Wang, Jianchao Wu, Minran Li, Xujing Liang, Jiaxiong Tan

**Affiliations:** ^1^ The First Affiliated Hospital of Jinan University, Guangzhou, China; ^2^ College of Pharmacy, Jinan University, Guangzhou, China; ^3^ Tianjin Medical University Cancer Institute and Hospital, National Clinical Research Center for Cancer, Tianjin, China; ^4^ Pediatric Oncology, Tianjin’s Clinical Research Center for Cancer, Tianjin, China

**Keywords:** tuberculosis, mycobacterium tuberculosis, immune checkpoint, CD8+ T cell, lymphocyte activation gene 3

## Abstract

**Background:**

Tuberculosis (TB) persists as a global health challenge, with its treatment hampered by the side effects of long-term combination drug therapies and the growing issue of drug resistance. Therefore, the development of novel therapeutic strategies is critical. This study focuses on the role of immune checkpoint molecules (ICs) and functions of CD8+ T cells in the search for new potential targets against TB.

**Methods:**

We conducted differential expression genes analysis and CD8+ T cell functional gene analysis on 92 TB samples and 61 healthy individual (HI) samples from TB database GSE83456, which contains data on 34,603 genes. The GSE54992 dataset was used to validated the findings. Additionally, a cluster analysis on single-cell data from primates infected with mycobacterium tuberculosis and those vaccinated with BCG was performed.

**Results:**

The overexpression of LAG-3 gene was found as a potentially important characteristic of both pulmonary TB (PTB) and extrapulmonary TB (EPTB). Further correlation analysis showed that LAG-3 gene was correlated with GZMB, perforin, IL-2 and IL-12. A significant temporal and spatial variation in LAG-3 expression was observed in T cells and macrophages during TB infection and after BCG vaccination.

**Conclusion:**

LAG-3 was overexpressed in TB samples. Targeting LAG-3 may represent a potential therapeutic target for tuberculosis.

## Introduction

An estimated over a quarter of the global population has been infected with mycobacterium tuberculosis (MTB) (Stuck et al., 2024). Despite the combinatorial anti-tuberculosis regimen based on Isoniazid (INH), Rifampicin (RIF), Pyrazinamide (PZA), and Ethambutol (EMB) has been curing numerous patients for the past fifty years. The significant long-term side effects of taking anti-tuberculosis medications have been overlooked and underestimated (Li et al., 2024). Challenges such as multidrug-resistant tuberculosis (TB) particularly multi-drug resistant (MDR-TB) and extensively drug-resistant (XDR-TB), TB patients with acquired immune deficiency syndrome (AIDS), and the rising number of potential MTB infections persisted as unresolved public health concerns (Li et al., 2024).

Promising efficiencies have been achieved in treating various solid tumors, such as melanoma, non-small cell lung cancer, and lymphoma, through immunotherapy strategies such as anti-programmed cell death 1/ligand 1 (PD-1)/(PD-L1) and anti-cytotoxic T-lymphocyte antigen 4 (CTLA-4) (Juric et al., 2024). These successes encouraged us to focus on the immune system restoration treatment strategies for TB patients. MTB is a smart and successful pathogen since it can persist in the intimidating environment of the host by taming and tuning the immune system. Dendritic cells (DCs) are one of the most important innate immune cells. Upon exposure to the MPT64 (Rv1980c) protein released by MTB, the differentiation of bone marrow-derived dendritic cells (BMDCs) into myeloid-derived suppressor cells (MDSCs), rather than dendritic cells (DCs), under a milieu conducive to DC differentiation (consisting of IL-4 and GM-CSF), was observed. This process led to the suppression of DC function through the upregulation of co-inhibitory molecules, including PD-L1, TIM-3, and Ly6C. Additionally, it impaired the migratory capacity of DCs by diminishing the expression of CCR7. Furthermore, this microenvironment facilitated the generation of regulatory T cells (Tregs) and concurrently limited the proliferation of both Th1 and Th17 cells (Chandra et al., 2022; Singh et al., 2022). The progression and outcomes of TB post-infection were largely determined by the function of immune cells, particularly NK cell and CD8+ T cells (Khayumbi et al., 2024; Reba et al., 2024). NK cell and CD8+ T cells typically eliminated pathogens and foreign antigens and established long-lasting immune memory through mechanisms such as granzyme B (GZMB)/perforin release, activation of the FAS/FASL system, and cytokine secretion (Khayumbi et al., 2024). In a TB mouse model, TB-specific CD8+ T cells exhibited impaired secretion of anti-TB factors like granzyme, perforin, IFN-γ, and TNF-α (Ben-Selma et al., 2011; Tousif et al., 2011). Studies on TB patient samples also have revealed varying levels of immune dysfunction in TB-specific CD8+ T cells (Lecocq et al., 2020; Qin et al., 2021). Furthermore, CD4+ T cells secreting IFNγ are essential for the intracellular bactericidal activity against Mycobacterium tuberculosis (Mtb), while CD4+ T cells producing IL-17 contribute to protective immunity against a variety of clinically relevant Mtb strains (Sefat et al., 2024). Therefore, restoring the anti-MTB immune function emerges as a promising avenue for anti-TB treatment.

Before 2007, researchers introduced the concept of T cell exhaustion in a mouse model of lymphocytic choriomeningitis virus (LCMV), identifying exhausted T cells by their heightened expression of various immunosuppressive receptors such as PD-1, CTLA-4, T cell immunoglobulin and mucin domain-3 (TIM-3) and Lymphocyte activation gene 3 (LAG-3) (Wherry et al., 2007; Kurachi, 2019; McLane et al., 2019). Immune checkpoint inhibitors can partially restore immune cell function by blocking the binding of ICs to their specific ligands (Kurachi, 2019). PD-1 has been widely recognized as a suppressive marker of CD8 and CD4 T cells in chronic viral infections in humans, including Human Immunodeficiency Virus (HIV), hepatitis B virus (HBV), and hepatitis C virus (HCV) (Fenwick et al., 2019; Zhou et al., 2023). Staphylococcus aureus (S. aureus) is the most common bacterial infection. Clinical Staphylococcus aureus inhibits human T-cell activity through interaction with the PD-1 receptor has been report (Mellergaard et al., 2023). Compared to healthy individuals (HI), the expression of PD-1 on CD4+ and CD8+ T cells in patients with TB is increased (Singh et al., 2013; Bandaru et al., 2014). Investigation by Tezera L.B proposed that hypoxia in TB lesions may contribute to the heightened expression of ICs (Tezera et al., 2020). Studies on TB mouse models revealed that mice lacking PD-1 exhibit poor response to MTB infection and escalated early mortality rates (Tousif et al., 2011). Blocking the PD-1/PD-L1 pathway can activate diverse immune cells (e.g., T cells, macrophages, dendritic cells) in TB granulomas (Tezera et al., 2020). Nevertheless, certain research indicated that terminally differentiated T cells post-TB infection do not express PD-1 as the exhausted receptor (Reiley et al., 2010; Woodworth et al., 2014; Boer et al., 2016). Initial research by Mardi C. Boer et al. indicated that killer cell lectin-like receptor subfamily-G1 (KLRG1) serves not only as a marker for terminal differentiation of TB-specific T cells in mouse models but also as an indicator of diminished human T cell proliferation following Bacilli Calmette-Guerin (BCG) vaccination (Boer et al., 2016). Jie Chen et al’s groundbreaking work unveiled that LAG-3 is overexpressed on CD4+ and CD8+ T cells in bronchoalveolar lavage fluid of TB patients, accompanied by reduced secretion of IFN-γ and granzyme B; furthermore, LAG-3 expression on CD8+ T cells correlates closely with the severity of TB (Chen et al., 2023). Our early investigations in leukemia revealed that ICs involved in antigen or pathogen immune evasion mechanisms are not singular, signifying potential differences in the expression patterns of ICs across diverse diseases and conditions (Huang et al., 2022; Zhao et al., 2022; Cai et al., 2023). Regrettably, current research on TB predominantly targets specific ICs, lacking comprehensive screening and studies encompassing samples from various ethnicities and multiple research centers.

This study encompassed a comprehensive analysis of the transcriptome databases GSE83456 and GSE54992, focusing on the 10 most prevalent ICs and 14 immune functional factors of CD8+ T cells in TB. It was found that the expression of LAG-3 gene was increased in TB group compared with the transcriptome data of normal people. Further correlation analysis showed that LAG-3 gene was associated with GZMB, perforin, IL-2 and IL-12 cytokines which related to CD8+T cell function. Subgroup analysis unveiled spatial and temporal variations in the expression of the LAG-3 gene in T cells and macrophages across various disease states subsequent to MTB infection, encompassing receipt of whether receiving anti-TB therapy, pulmonary and extrapulmonary TB, and diverse time points post-BCG vaccination. In summary, this research aims to pinpoint potential ICs that modulate the cytotoxic activity of CD8+ T cells in TB patients by systematically analyzing multi-center TB databases, thereby providing more reference for anti-TB immunotherapy targeting the reversal of CD8+ T cell immune function.

## Methods

### Publicly available datasets

In this study, publicly available datasets were utilized. Specifically, the GSE83456 dataset was downloaded from the Gene Expression Omnibus (GEO, https://www.ncbi.nlm.nih.gov/geo/) database, which includes gene expression profiles and clinical information obtained using RNA-Seq and microarray technologies (Li et al., 2023). Additionally, the GSE54992 TB dataset was employed as a validation set. The above two GEO data are human peripheral blood derived transcriptome data. Furthermore, single-cell sequencing data of TB from the SCP257, SCP1749, and SCP796 studies were analyzed after being processed through the Single cell portal (https://singlecell.broadinstitute.org/). The single cell sequenced tissue was derived from the granulomatous tissue of TB-infected primate.

### Identification of TB specific ICs

In this study, we first utilized the R package “limma” to perform differential expression genes (DEGs) analysis on 34,603 genes from 92 TB patient samples, including 47 cases of extrapulmonary TB (EPTB) and 45 cases of pulmonary TB (PTB), as well as 61 HI samples from the GSE83456 dataset. By integrating the results of differential gene expression analysis with TB-related literature, we selected 10 ICs that are potentially associated with the immune evasion mechanisms in TB patients for subgroup analysis. These molecules include PDCD1, LAG-3, CTLA-4, IDO-1, BTLA, CD47, TIM-3, TIGIT, VISTA, and CD276, aiming to identify ICs that may be specifically expressed in TB samples. We further used the differential expression of the IFN-γ gene to select ICs with high specificity to TB. Finally, by combining clinical diagnostic data, we constructed Receiver Operating Characteristic (ROC) curves, and assessed the specificity of the selected ICs by calculating the Area Under the Curve (AUC).

### The relation between TB-specific ICs and the function of CD8+ T cells

To delve into the correlation between TB-specific ICs and the immune function of CD8+ T cells, we initiated with an intergroup differential analysis to compare TB patients with HI regarding three core functions of CD8+ T cells in anti-pathogen immunity: granzyme/perforin, FAS/FASL, and cytokine secretion. Following this, based on the candidate molecules previously identified, we ranked all TB samples, and divided them into high and low expression groups using the median as the cutoff point, to further compare the differences in genes related to CD8+ T cell function between the two groups. By combining the results from these two rounds of intergroup differential analyses, we were able to preliminarily reveal the potential mechanisms by which TB-specific ICs impact the immune function of CD8+ T cells. Ultimately, through a detailed correlation analysis between genes that differ in CD8+ T cell immune function and TB-specific ICs, we aimed to clarify their relationship.

### Analysis of immune cell infiltration

All data analyzed in this study were derived from the GSE83456 dataset. The method used for cell-type identification was CIBERSORT, which estimates relative subsets of RNA transcripts (Chen et al., 2018). The CIBERSORT analysis was conducted using the R statistical programming language with the CIBERSORT package installed via the devtools framework. The analysis required two input files: a gene expression matrix specific to our study samples and the LM22 signature file, which contains the gene expression signatures of the 22 immune cell subsets. Initially, the difference in immune cell subset infiltration between the TB group and the HI group was examined. Subsequently, the disparities in immune infiltration between the candidate gene high-expression group and low-expression group, as well as between the IFN-γ high-expression group and low-expression group, were explored. In conclusion, the most pertinent immune cell subsets associated with the candidate IC genes were identified through matching analysis.

### Validation of tuberculous specific ICs

The RNA dataset from GSE54992, which includes profiles from six HI, six potentially infected individuals, and nine TB patients also encompassing data from the same nine TB patient post-anti-TB treatment were utilized for the validation of component analysis.

### Single cell validation in primates

After retrieving the data for SCP257 and SCP1749 items through the Single Cell Portal, we compared the cluster analysis results of TB-specific ICs at 4 weeks and 10 weeks post-infection. The objective was to cross-validate the findings from the GSE83456 dataset. Additionally, data from the SCP796 project were obtained via the portal to examine the variation in distribution of TB-specific ICs 13 and 25 weeks after BCG vaccination, serving as a supplementary verification of the impact of TB antigens on ICs. This segment of the analysis was conducted on the following website: https://singlecell.broadinstitute.org/.

### KEGG pathway enrichment analysis

The DEGs between TB patients and HI were extracted from the GSE83456 dataset. Subsequently, the gene symbols in the list were converted to their corresponding KEGG database IDs. The entire KEGG enrichment analysis was conducted on the online platform DAVID (Database for Annotation, Visualization and Integrated Discovery) following the ID conversion process.

### Statistical methods

The R software (version 4.2.1) was utilized for the statistical analysis and graphing. For comparing differences between two groups, we employed the two-tailed unpaired Student’s t-test and the Wilcoxon test. Differences in two groups of qualitative variables were compared by the Fisher’s exact test. The correlation analyses in this study were all conducted using the Pearson product-moment correlation coefficient to evaluate the linear relationship between variables. The value of the correlation coefficient ranges from -1 to 1, indicating the strength and direction of the linear relationship between the variables. To determine whether the correlation is statistically significant, we performed a two-tailed t-test. All the differences were considered statistically significant with *P* < 0.05.

## Result

### LAG-3 was found to be overexpressed in the tuberculosis group

**Figure 1:**
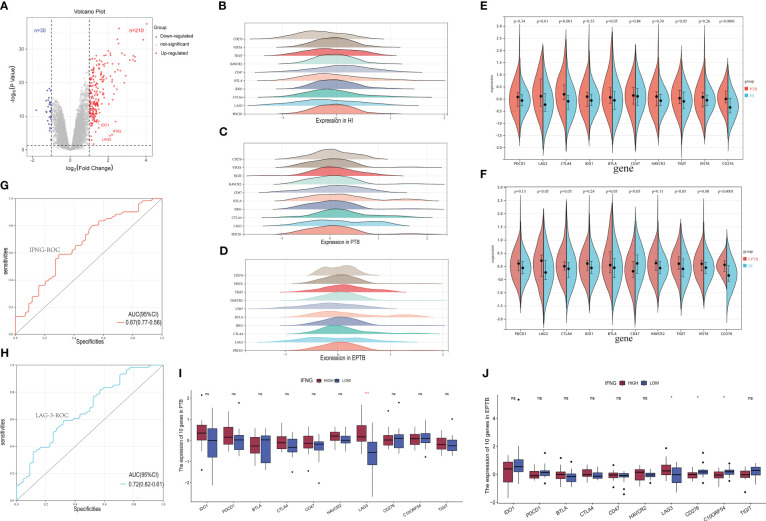
Screening of specific immune checkpoint molecules for tuberculosis infection. **(A)**. The volcano plot for differentially expressed genes (DEGs) (FC > 1.5 and adjusted *p* < 0.05), and the red, gray and blue circles indicate up- regulated, stable expressed and down-regulated of TB DEGs, respectively. **(B–D)**. Expression patterns of 10 immune checkpoint genes in HI, PTB and EPTB subgroups. The horizontal coordinate is the level of gene expression after normalization and standardization, and different colors correspond to different molecules. **(E, F)**. Multiple immune checkpoint molecules were analyzed in subsets of PTB, EPTB, and HI. Blue for HI, red for TB sample, p< 0.05 was considered statistically significant. **(I, J)**. The TB samples were divided into high expression group (red column) and low expression group (blue column) according to IFNG sequence, and then the expression differences of immune checkpoint molecular genes were analyzed. **(G, H)** It is the specific ROC curve of IFNG (red curve) and LAG-3 (blue curve) in tuberculosis, and the corresponding AUC values are 0.67 and 0.72, respectively. ns means no statistical significance, *p < 0.1; **p < 0.05; ***p < 0.01; ****p < 0.001, respectively.

In this study, a comprehensive analysis was conducted to identify differentially expressed genes between TB patients and HI. A total of 92 samples of TB infections (47 cases of EPTB and 45 cases of PTB) and 61 HI samples from the GSE83456 dataset, covering 34,603 genes, were included. Differential gene expression analysis revealed 210 significantly up-regulated genes, including IDO1, LAG-3, and IFN-γ, and 30 significantly down-regulated genes (log2-fold change was 1.5, P-value less than 0.05 was considered significant), showed as Figure 1A. We demonstrated significant variances in gene expression between the TB group and the HI group, whereas the expression profiles exhibited a high degree of similarity between the PTB and EPTB groups (**Figures 1B-D**). Specifically, the PTB group exhibited significant upregulation in LAG-3, CTLA-4, BTLA, TIGIT, and CD276. As shown in **Figures 1E, F**, higher expressions of CD47 and VISTA were observed in the EPTB group compared to the HI group. PTB and EPTB samples were then stratified based on IFN-γ gene expression levels to conduct differential analysis of ICs. In **Figures 1I, J**, a significant increase in LAG-3 gene levels was observed in the IFN-γ high-expression group of PTB samples as well as elevated levels of LAG-3, CD276, and VISTA in the high IFN-γ group of EPTB samples. Furthermore, according to **Figures 1G, H**, LAG-3 was demonstrated superior efficacy in TB identification compared to IFN-γ (AUC was 0.72 and 0.67).

### LAG-3 may be related to impaired CD8+T cell immune function in TB

**Figure 2:**
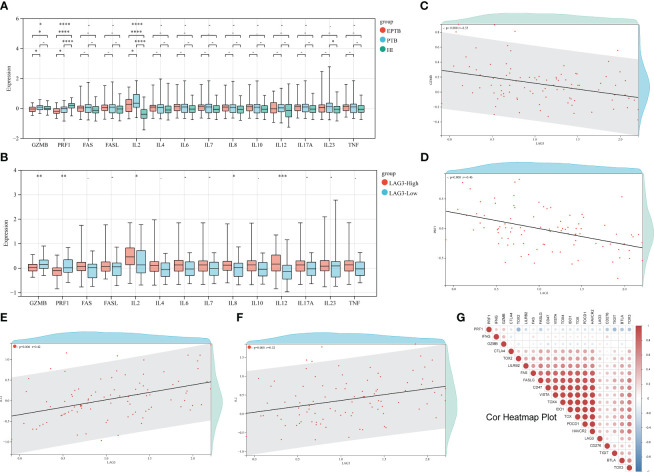
Relationship between LAG-3 and CD8+T cell immune function genes. **(A)**. First, the differences of 14 CD8+T cell-related immune function genes between TB and HI were analyzed. The green columns are HI group, the blue columns are PTB, and the red columns are EPTB. **(B)**. The difference of immune function related genes between the LAG-3 high expression group and the low expression group was compared. The red column was the LAG-3 high expression group, and the blue column was the low expression group. **(C–F)**. Correlation analysis of GZMB, PRF1, IL-2 and IL-12 and LAG-3, respectively, with each red dot representing 1 TB sample. **(G)**. A heat map of the correlation between the remaining immune checkpoints and some of the immune functional genes, with negative correlations in blue and positive correlations in red. ns/- means no statistical significance, *p < 0.1; **p < 0.05; ***p < 0.01; ****p < 0.001, respectively.

The correlation heatmap displayed in **Figure 2G** illustrated the overall association between various ICs and genes related to CD8+ T cell immune function. A significant decrease in the levels of GAMB and perforin genes in all TB patients compared to the HI group was demonstrated (**Figure 2A**). In addition, EPTB patients exhibited lower levels of these genes compared to PTB patients. Moreover, subgroup analysis revealed elevated IL-2 gene expression in TB patients, while IL-23 levels in PTB patients surpassed those of the HI group. Conversely, no significant differences were observed in genes related to the FAS/FASL axis as depicted in **Figure 2A**. Next step is to further explore the correlation between LAG-3 expression and CD8+ T cell immune function. Notably, lower levels of GZMB and perforin genes were discovered in the LAG-3-high group, while cytokine such as IL-2, IL-8, and IL-12 were higher (**Figure 2B**). Subsequent correlation analyses indicated a negative relationship between LAG-3 expression and GZMB and perforin genes, along with positive correlations with IL-2 and IL-12 (**Figures 2C-F**, *P*>0.05).

### Differential expression of LAG-3 and CD8+T cell functional genes

To further investigate the influence of ethnicity, age, and disease status on LAG-3 gene expression and CD8+ T cell immune function, a detailed subgroup comparative analysis was conducted (refer to **Supplementary Figures 2A-I**). In the HI group, we observed that the LAG-3 mRNA levels in Black individuals were significantly higher than in White (**Supplementary Figure 2B**). However, in TB patients, the LAG-3 gene expression levels in White were significantly higher than in other races (**Supplementary Figure 2C**), the phenomenon not observed in the subgroup analysis of PTB patients (**Supplementary Figure 2D**). Subgroup analysis stratified by age revealed that although there were no significant differences in LAG-3 gene expression among TB patients in different age groups, younger patients (under 60 years old), tended to have higher levels of GZMB and PRF1 mRNA, while older patients (over 60 years old) had relatively higher IL-2 levels (**Supplementary Figures 2E-I**).

### LAG-3 expression was associated with plasma cell and T cell infiltration in TB

**Figure 3:**
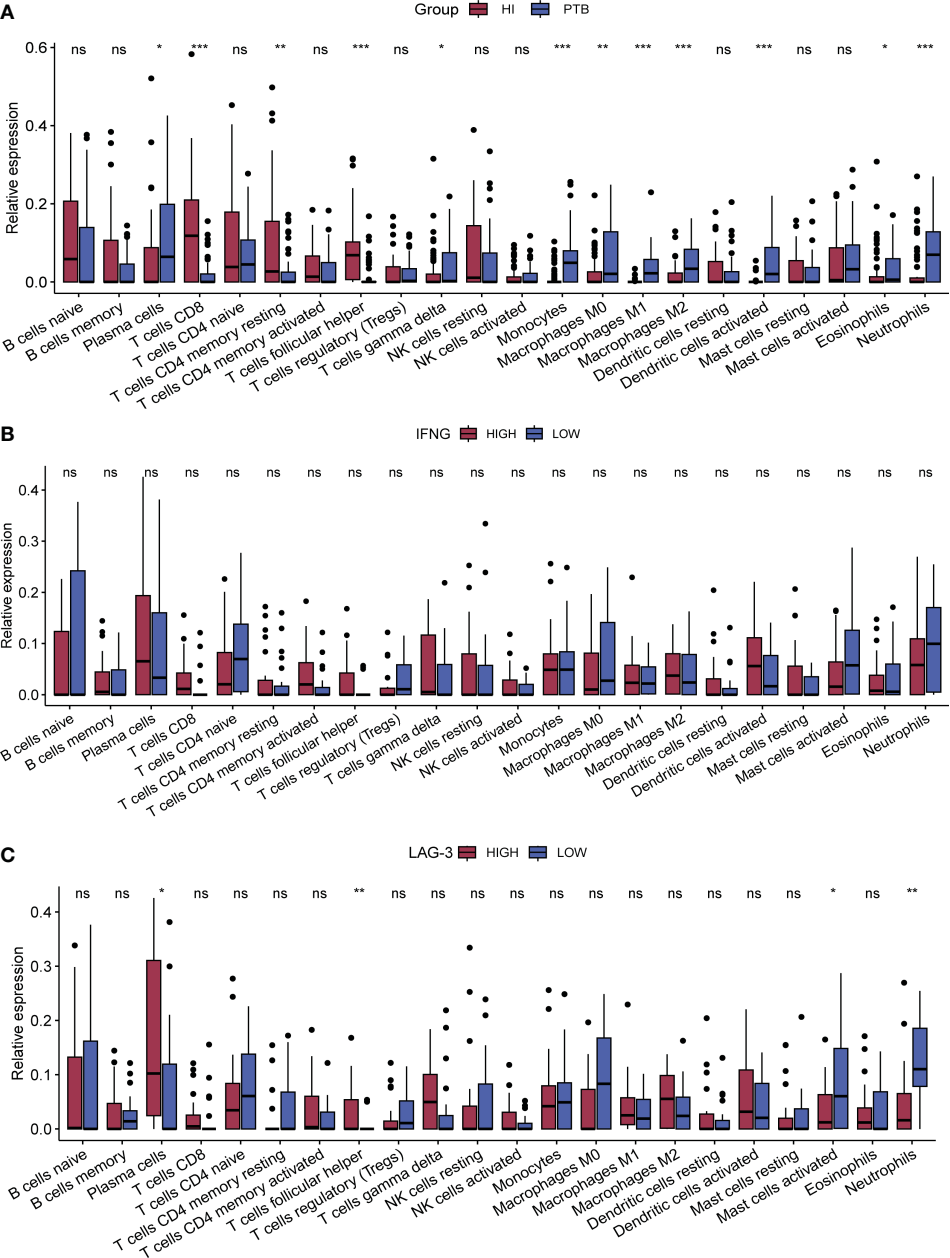
Analysis of immune-infiltration between subgroups. CIBERSORT was used for all subgroup immune-cell infiltration analyses. **(A)**. The difference of immune cells between PTB and HI was compared. The red column was HI group and the blue column was PTB group. **(B, C)**. Immune cell infiltration between subgroups of IFNG and subgroups of LAG-3 in TB samples, respectively. The red column in B represents the group with high expression of IFNG and the blue column represents the group with low expression of IFNG, while the red column represents the group with high expression of LAG-3 and the blue column represents the group with low expression of LAG-3. *p < 0.1; **p < 0.05; ***p < 0.01; respectively. ns means no statistical significance.

Displayed in **Figure 3A**, it illustrated that PTB patients had higher counts of neutrophils, plasma cells, monocytes, dendritic cells (DC), macrophages (including M0, M1, and M2 subtypes), and γδ (GD) T cells compared to the HI group, whereas the numbers of CD8+ T cells, memory CD4+ T cells, and auxiliary follicular T cells were relatively lower. Despite lacking statistical significance, an increasing trend in the infiltration of CD4+ T cells, CD8+ T cells, GD T cells, and natural killer (NK) cells was observed in the high-IFN-γ group (**Figure 3B**). Depicted in **Figure 3C**, TB patients with high LAG-3 expression exhibited elevated levels of plasma cells, CD8+ T cells, activated memory CD4+ T cells, and auxiliary T follicular cells. Conversely, in the TB group with low LAG-3 expression, neutrophil infiltration was notably pronounced.

### Verification results of the GSE54992 dataset

**Figure 4:**
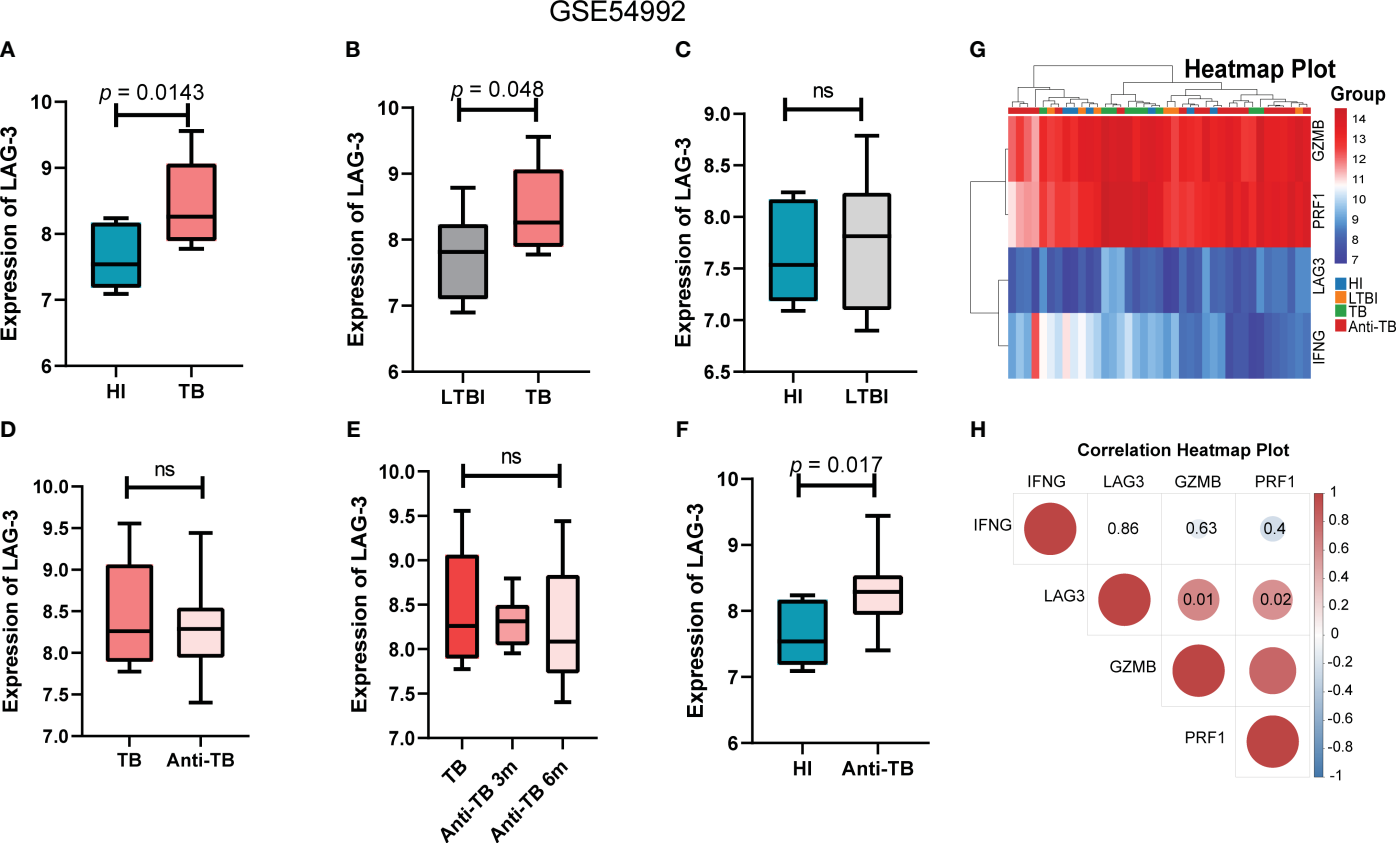
LAG-3 was identified as a TB-specific immune checkpoint in GSE54992 database. **(A–C)**. Intergroup difference analysis of LAG-3 in HI, LTBI and TB. Blue columns, gray columns and red columns represent HI, LTBI and TB respectively. **(D–F)**. The expression differences of LAG-3 between TB samples before and after treatment and at different time points after treatment were analyzed. The dark red columns, light red columns, and blue columns represent untreated, antituberculosis treated samples, and normal controls, respectively. **(G–H)**. Shows the correlation heat map between IFNG, LAG-3, GZMB and PRF1. Blue represents negative correlation and red represents positive correlation. The above results are from the validation set GSE54992 database. ns means no statistical significance.

Utilizing another independent TB dataset GSE54992 as a validation set, we corroborated the differential expression of the LAG-3 gene in TB patients. Analysis of the validation set data revealed reduced levels of GZMB and PRF1 in latent tuberculosis infection (LTBI) and TB patients compared to those in HI, alongside significantly elevated expression levels of the LAG-3 and IFN-γ genes in these samples. Notably, post anti-TB drug treatment, the gene expression profile of patients gradually aligned with that of the HI samples (**Figure 4G**). The results of the intergenic correlation analysis were depicted in **Figure 4H** using a correlation heatmap. The LAG-3 gene levels in the TB group were markedly founded higher than those in the HI and LTBI groups, with no significant difference observed between the LTBI and the HI group (**Figures 4A-C**). This observation was in line with our previous findings from the analysis of the GSE83456 dataset. Delving further into the impact of anti-TB treatment on LAG-3 gene levels suggested a gradual decrease in patient samples over the course of treatment duration, indicating a declining trend (**Figures 4D, E**). Notably, even throughout anti-TB treatment, the LAG-3 levels in TB patients remained elevated compared to those in the HI group, highlighting the potential of LAG-3 as a sensitive biomarker during treatment (**Figure 4F**).

### The spatiotemporal relationship between LAG-3 and TB infection was verified at the single-cell level

**Figure 5:**
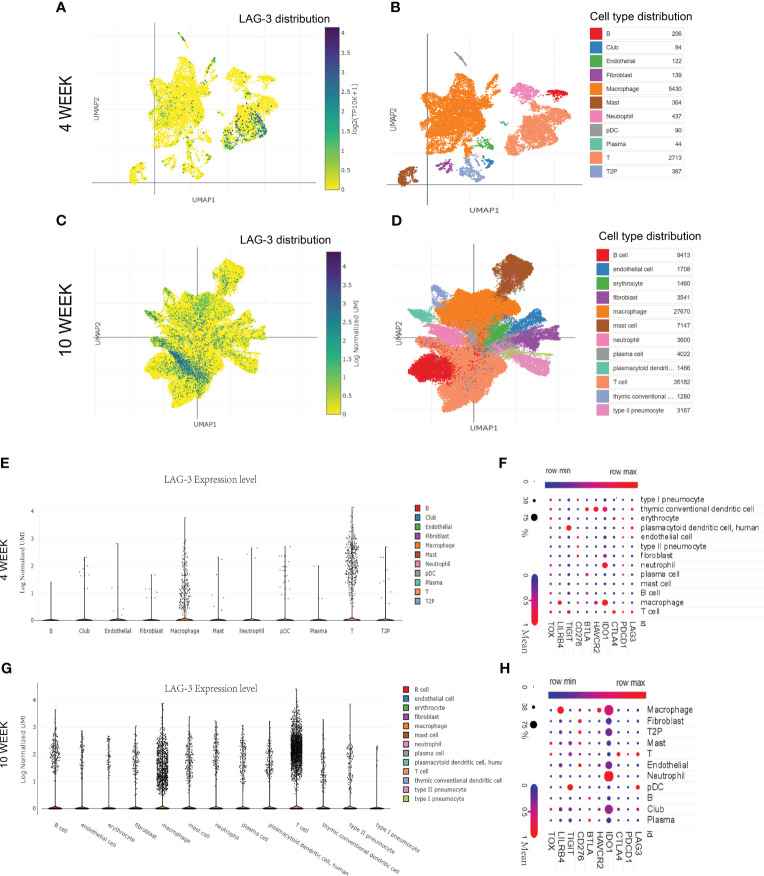
The distribution of LAG-3 with the duration of TB infection. **(A, B)**. Single cell cluster analysis of granuloma after 4 weeks of TB infection. The blue dots represent the distribution of LAG-3, and the darker the color, the higher the density, while the specific positions correspond to different immune cells represented by different colors in the B diagram. **(C, D)**. Single cell cluster analysis of granuloma after 10 weeks of TB infection. **(E–G)**. The expression levels of LAG-3 in various immune cell subsets after 4 and 10 weeks of TB infection were shown, respectively. **(F–H)**. Shown are correlated heat maps of multiple immune checkpoint molecules and immune cell subsets. The change in color is the mRNA mean, while the size of the dots represents the density (percent). All of the above analyses were performed at the single-cell portal.

This study delved deeper into investigating the impact of the duration of MTB infection on LAG-3 expression and its distribution in immune cells. The SCP257 and SCP1749 studies in the public single-cell database were analyzed, encompassing high-throughput single-cell mRNA sequencing of granulomas in non-human primates at four and ten-weeks post-infection, respectively. It demonstrated a significant increase in LAG-3 mRNA levels after ten weeks of infection compared to samples infected for 4 weeks, particularly expressed in T cell subpopulations (**Figures 5A, B**). Clustering analysis of multiple immune cell subpopulations revealed a progressive rise in the distribution density of LAG-3 in T cell subpopulations with prolonged infection time (**Figures 5A-D**). A more detailed subpopulation analysis showed a noteworthy elevation in LAG-3 mRNA levels after 10 weeks of infection in various immune cell subpopulations, including plasma cells, T cells, and macrophages, as opposed to early infection. The association between other ICs and immune cell subpopulations was depicted in **Figures 5F, H**.

### The spatiotemporal relationship between BCG injection and LAG-3

**Figure 6:**
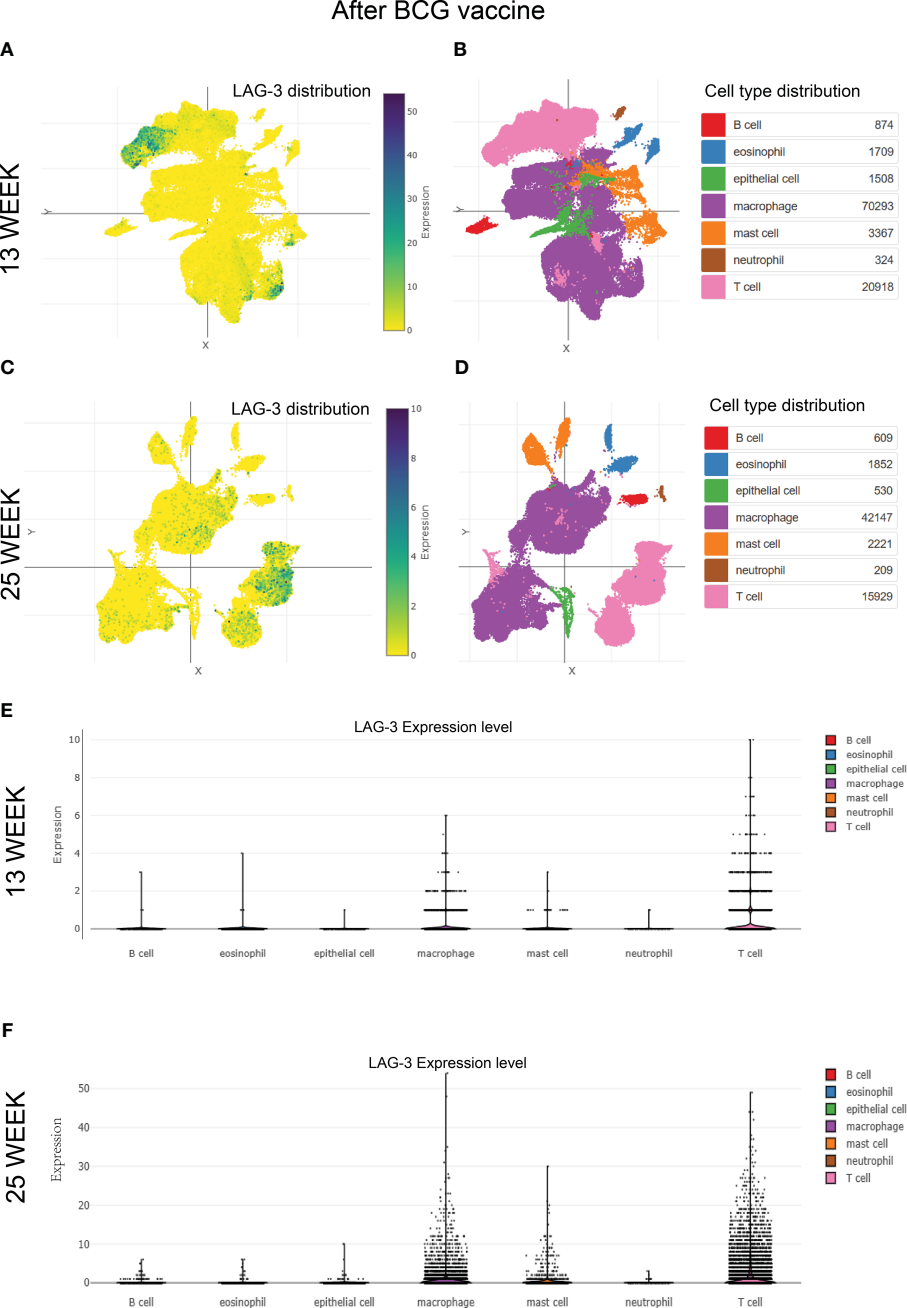
The distribution of LAG-3 after BCG vaccine injection. **(A–D)**. Single-cell cluster analysis of LAG-3 distribution in immune cell subsets after 13 and 25 weeks of BCG vaccine injection, respectively. The blue dots represent the distribution of LAG-3, and the darker the color, the higher the density, while the specific positions correspond to different immune cells represented by different colors in the **(B, D)** diagram. **(E, F)**. The expression levels of LAG-3 in various immune cell subsets after 13 and 25 weeks of BCG vaccine injection, were shown, respectively. All of the above analyses were performed at the single-cell portal.

Given the crucial role of the BCG vaccine in global TB control, this study delved into the detailed exploration of the dynamic changes in LAG-3 expression after BCG vaccination. Analysis of single-cell mRNA sequencing data from non-human primate samples vaccinated with BCG at 13 and 25 weeks revealed a predominant concentration of LAG-3 expression in T cell and macrophage populations at both time points (refer to **Figures 6A-D**). Comparative analysis indicated a significant increase in the distribution density of LAG-3 in samples collected 25 weeks post-BCG vaccination compared to those at 13 weeks, with a distinct concentration trend observed in T cell subpopulations (**Figures 6A-D**). Subsequent subgroup analysis verified that a prolonged duration of BCG vaccination led to a gradual increase in LAG-3 gene expression levels, primarily concentrating this elevation in T cell and macrophage subpopulations (**Figures 6E, F**).

### KEGG pathway enrichment analysis

**Figure 7:**
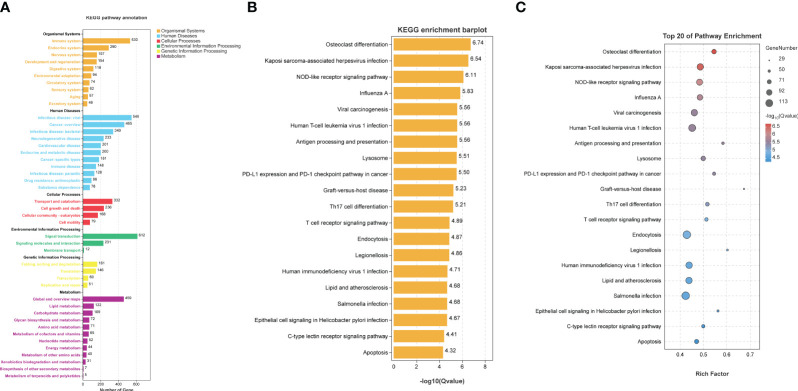
KEGG pathway enrichment analysis. KEGG pathway enrichment analysis was based on TB DEGs from GSE83456 database. **(A)** Preliminary enrichment of possible pathways from six regions, including human systems, human diseases, and cellular processes. **(B, C)** presents the top 20 possible relevant signaling pathways.

In the concluding phase of our research, we conducted a KEGG pathway enrichment analysis on the DEGs between TB patients and HI in the GSE83456 dataset. It illustrated that the DEGs primarily associated with various pathways related to infectious diseases and the human immune system, with the most significantly enriched top 20 pathways depicted in **Figures 7A, B**. The top three pathways among these included osteoclast differentiation, Kaposi’s sarcoma-associated herpesvirus infection, and NOD-like receptor signaling pathways as shown in **Figure 7C**. Additionally, **Supplementary Figure 3** presented a schematic diagram of specific signaling pathways linked to tuberculosis.

## Discussion

After the discovery of antigen-specific CD8+ T cell exhaustion in a prolonged chronic virus infection model, it garnered increased attention in various infectious diseases and tumor research (Wherry et al., 2007). Exhausted CD8+ T cells exhibited heightened expression of ICs such as PD-1, CTLA-4, TIM-3, and LAG-3, concomitant with adynamic T cell cytotoxicity and pathogen clearance capabilities (Huang et al., 2019; Tan et al., 2020). Blocking these ICs to restore partial T cell immune function emerged as a highly appealing strategy for anti-infection and anti-tumor therapies (Singh et al., 2013; Cai et al., 2023). The favorable therapeutic outcomes of anti-PD-1/PD-L1 and anti-CTLA-4 in solid tumor treatment underscored the substantial research potential of this approach in infectious diseases, notably TB infection (Juric et al., 2024).

PD-1 was widely recognized as an inhibitory marker on CD8 and CD4 T cells in chronic viral infections in humans, such as chronic HIV, HBV, and HCV infections (Fenwick et al., 2019; Zhou et al., 2023). Staphylococcus aureus (S. aureus) is the most common bacterial infection. Clinical Staphylococcus aureus inhibits human T-cell activity through interaction with the PD-1 receptor; this has been report. This findings suggest that therapeutically targeting PD-1 is a possible future strategy for treating certain antibiotic-resistant staphylococcal infections (Mellergaard et al., 2023). The expression of PD-1 on CD4+ and CD8+ T cells was higher in TB patients compared to HI (Singh et al., 2013; Bandaru et al., 2014). Tezera L.B ‘s studies suggested that hypoxia within TB lesions may have contributed to the upregulation of ICs expression (Tezera et al., 2020). Experiments using a TB mouse model demonstrated that mice deficient in PD-1 exhibited a poor response to MTB infection and an increased early mortality rate (Tousif et al., 2011). As ligand for PD-1, PD-L1 and PD-L2, expressed on a variety of immune cells, play multiple regulatory roles in the host immune response to Mycobacterium tuberculosis infection (Yu et al., 2024). In addition, during MTB infection, PD-1/PD-L1, PD-L2 signaling is also involved in the host inflammatory response, as well as the potential roles of PD-1/PD-L1, PD-L2 in the diagnosis and treatment of tuberculosis (Yu et al., 2024). Inhibiting the PD-1/PD-L1 pathway could activate various immune cells, including T cells, macrophages, and dendritic cells, within TB granulomas (Tezera et al., 2020). Contrary to some studies, exhaustion receptors observed on terminally differentiated T cells post-TB infection were not PD-1 but other receptors (Boer et al., 2016; Chen et al., 2023). In our research, we initially conducted a comparative analysis of the expression of 10 ICs related to T cell exhaustion in the gene expression profiles of TB patients and HI. Our findings aligned with most established TB research, showing distinct expression patterns of multiple ICs, such as LAG-3, CTLA-4, TIGIT, VISTA, CD47, and CD276, between TB patients and HI, while the expression profiles of ICs in PTB versus extrapulmonary TB were nearly identical.

In this study, we aimed to finding IC molecules that express differences in TB samples and analyze their impact on the immune function of CD8+ T cells. TB-specific IFN-γ release served as a vital marker for distinguishing TB from LTBI and HI (Sudbury et al., 2020). Our analysis found that the LAG-3 gene was overexpressed in the TB group compared to the HI group. Further correlation analysis of TB sample data showed that LAG-3 was correlated with GZMB, perforin IFN-γ, and some interleukins. It was noted in previous research that chronic infection from prolonged exposure to pathogens resulted in sustained expression of immune inhibitory receptors like LAG-3 on CD4+ T and CD8+ T cells (Chen et al., 2023). Activation of the LAG-3 pathway transmitted inhibitory signals that hindered complete activation of CD8+ T cells (Qin et al., 2021). Additionally, Jie Chen et al. observed increased LAG-3 expression in CD4+ and CD8+ T cells of TB patients, linking this upregulation to functional impairments in CD8+ T lymphocytes and the severity of TB (Chen et al., 2023). Further experiments with co-culturing anti-LAG-3 with peripheral blood mononuclear cells from TB patients verified that inhibition of LAG-3 led to elevated GZMB expression in peripheral blood CD8+ T cells. These outcomes aligned with our discovery of reduced expression of GZMB and perforin genes in TB samples with high LAG-3 expression. Our finding confirmed that lower levels of GZMB and perforin genes in EPTB cases and individuals over 60 years old. Notably, our study found a positive correlation between IL-2 and IL-12 expression and LAG-3 gene expression, contrasting with Jie Chen et al. who reported decreased IL-2 secretion by LAG-3+ CD4+ T cells (Chen et al., 2023). Noteworthy from a mouse model, prolonged exposure to TB antigens resulted in reduced IL-2 secretion compared to short-term exposure, indicating a relationship between IL-2 levels and the duration of TB infection (Liu et al., 2019). Furthermore, the polymorphism and genetic variations of numerous cytokines such as IL-17F, TNF-α, and IL-10 have also been found to be closely associated with susceptibility to TB and resistance to anti-TB treatment (Ben-Selma et al., 2011; Peng et al., 2013). Nonetheless, research on the genetic polymorphisms of IL-2 and IL-12 in TB patients was still lacking. There might have been disparities in LAG-3 gene expression among different racial groups in TB and non-TB conditions. Although multicenter studies on tumor immunotherapy did not discern substantial racial discrepancies, further investigation was warranted to unravel potential racial differences in the genetic profile of ICs.

Finally, we investigated the variations in LAG-3 expression over time and location after TB infection. The single-cell clustering analysis conducted in non-human primate TB models revealed an elevated accumulation of LAG-3 on T cells and macrophages as TB infection and BCG injection duration extended, aligning with the outcomes from our subcomponent immune infiltration analysis (**Figures 3A-C**, **5**, **6**). A separate study indicated a relationship between the interaction of macrophages and CD8 T cells in bronchoalveolar lavage and latent TB infection (Yang et al., 2023). The Monocyte macrophage (MM)-CCL23 pathway primarily attracted CD8-CCR6 T cells through the CCL20/CCR6 axis, a key feature associated with protective immunity in latent TB infection (Yang et al., 2023). Boer MC et al. observed that BCG vaccination induced KLRG1 expression on BCG-stimulated CD8+ T cells, leading to a significant reduction in proliferation (Boer et al., 2016). These findings will further promote our attention to the dynamic changes of ICs in various immune cells over time and space, as well as focus more on the interactions between immune cells.

Although another dataset GSE54992 was used for validation, this study still had certain limitations. Firstly, the main content of that research was solely based on gene-level analysis without considering the impact of gene transcription, translation, and epigenetic modifications. The lack of *in vitro* and preclinical animal model experiments to further validate our current study is another limitation of this study. Additionally, the insufficient sample size might have affected the rigor of the research results. It is important to note that, despite multiple correlations being analyzed, no *post-hoc* correction was applied. This omission may significantly increase the risk of type I errors. Finally, a real-world clinical large-sample validation and cellular functional experiments were needed to be supplemented in our subsequent studies.

## Conclusion

The overexpression of LAG-3 gene was potentially an important feature of PTB and EPTB. Further correlation analysis showed that LAG-3 gene was correlated with GZMB, perforin, IL-2 and IL-12. A significant temporal and spatial variation of LAG-3 in T cells and macrophages was exhibited during TB infection and after BCG vaccination. Targeting LAG-3 to Restore CD8+ T Cell Cytotoxicity may be a new approach to the treatment of TB.

## Data availability statement

The original contributions presented in the study are included in the article/**Supplementary Material**. Further inquiries can be directed to the corresponding authors.

## Ethics statement

Ethical approval was not required for the study involving humans in accordance with the local legislation and institutional requirements. Written informed consent to participate in this study was not required from the participants or the participants’ legal guardians/next of kin in accordance with the national legislation and the institutional requirements.

## Author contributions

JT: Writing – original draft, Writing – review & editing, Conceptualization, Data curation, Formal Analysis, Investigation, Methodology, Resources, Validation. YP: Conceptualization, Formal Analysis, Writing – original draft, Writing – review & editing. ZY: Investigation, Software, Supervision, Validation, Writing – original draft, Writing – review & editing. LH: Conceptualization, Data curation, Investigation, Methodology, Supervision, Writing – original draft, Writing – review & editing. MX: Formal Analysis, Project administration, Writing – original draft, Writing – review & editing. RC: Data curation, Resources, Visualization, Writing – original draft, Writing – review & editing. DL: Formal Analysis, Project administration, Software, Writing – original draft, Writing – review & editing. XW: Project administration, Validation, Writing – original draft, Writing – review & editing. JW: Methodology, Supervision, Conceptualization, Investigation, Writing – original draft, Writing – review & editing. ML: Writing – original draft, Writing – review & editing, Conceptualization, Funding acquisition, Investigation. XL: Writing – original draft, Writing – review & editing, Funding acquisition, Investigation.

## Supplementary material

Supplementary Figure 1 Sample information distribution in GSE83456 database.

Supplementary Figure 2 The distribution of LAG-3 in different disease states, ethnic groups and age groups in GSE83456 database.

Supplementary Figure 3 Map of global signaling pathways associated with TB disease immunity.
