# Supplementary figures and images for: Overexpression of LAG-3: a potential indicator of low immune function in tuberculosis
Source: Front Cell Infect Microbiol. 2024 Jun 18;14:1410015. doi: 10.3389/fcimb.2024.1410015 (PMC11217189; doi:10.3389/fcimb.2024.1410015)

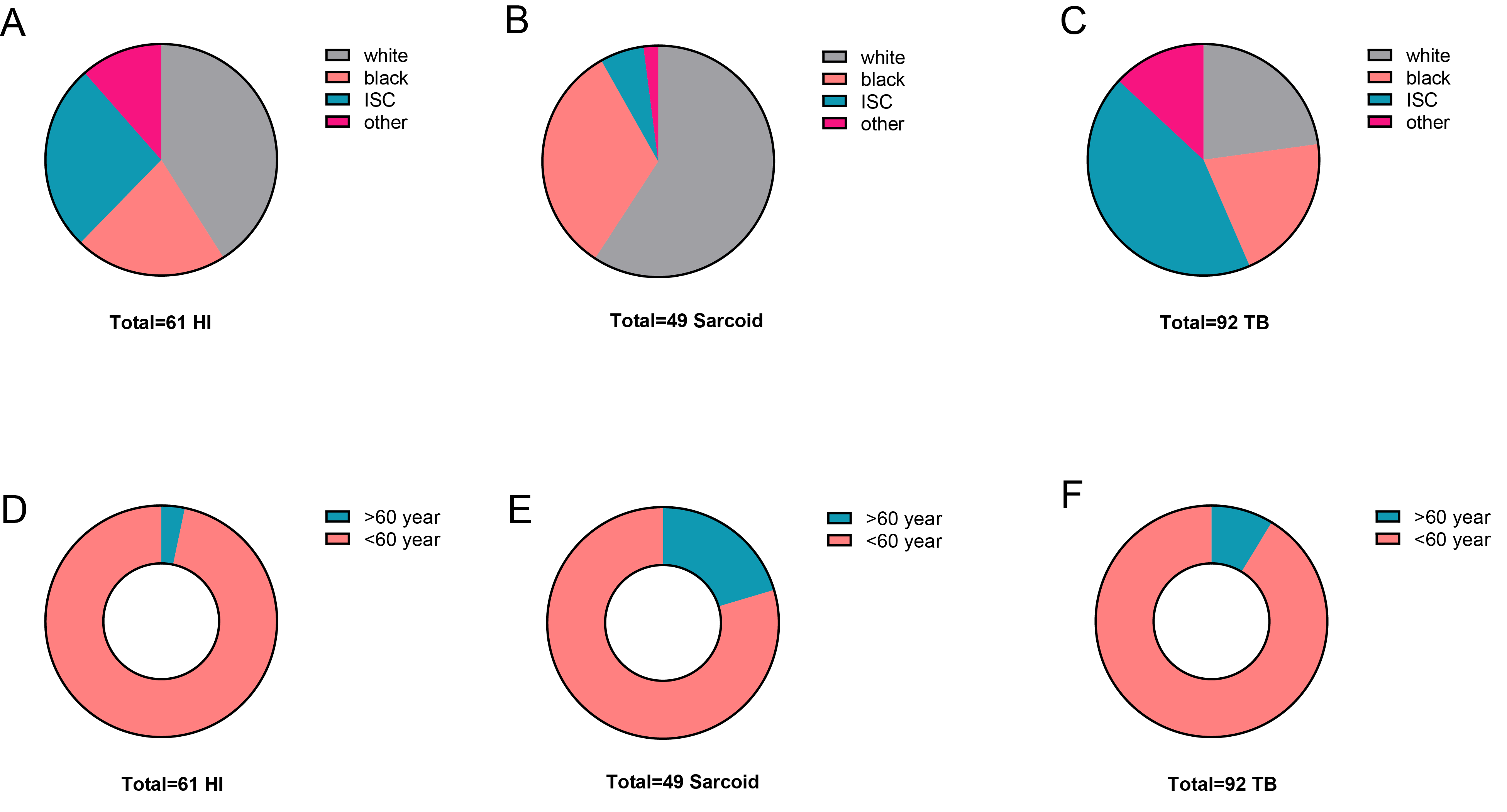

Supplement: Supplementary Figure 1 — Sample information distribution in GSE83456 database. [file Image_1.tif]

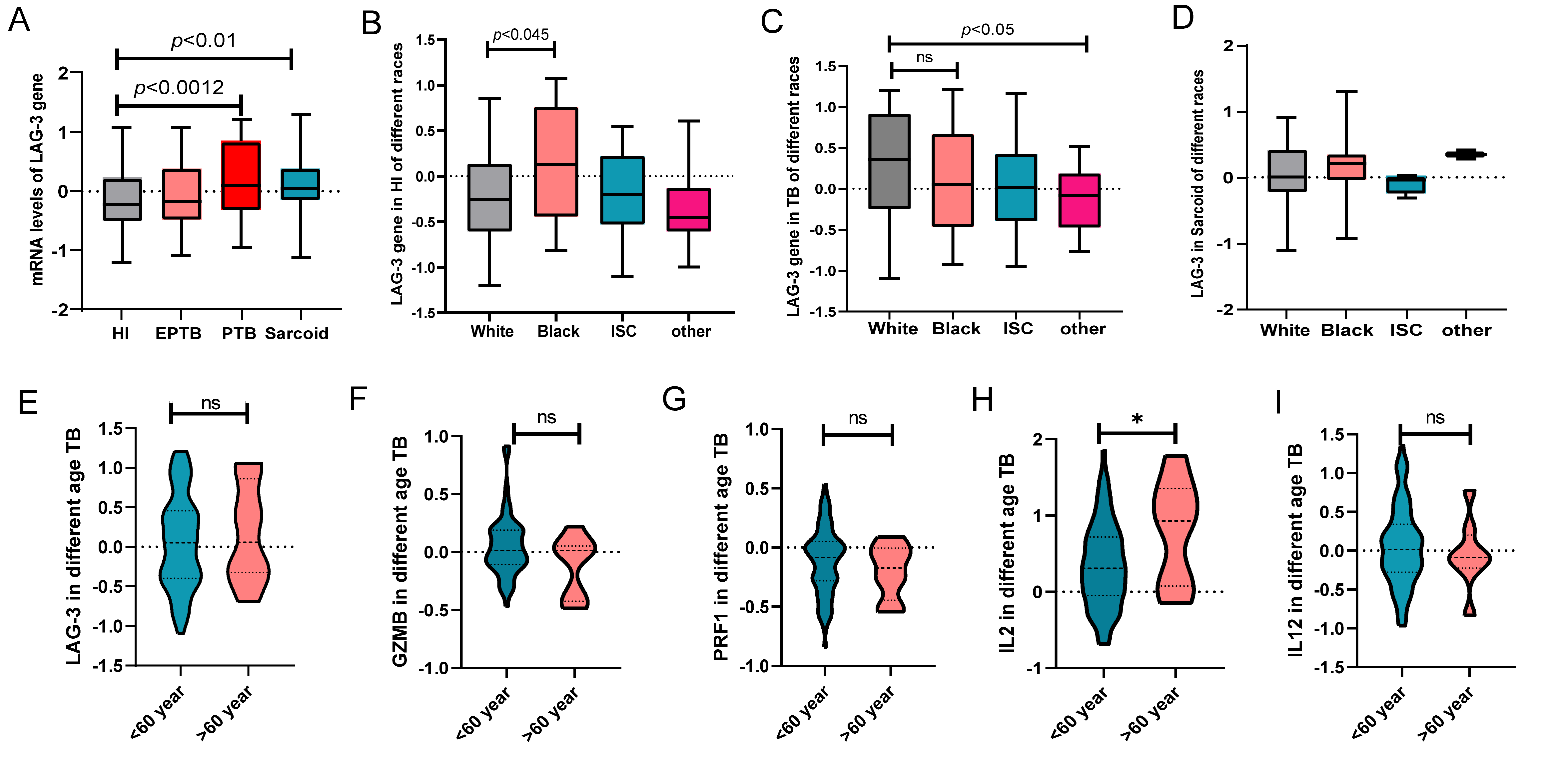

Supplement: Supplementary Figure 2 — The distribution of LAG-3 in different disease states, ethnic groups and age groups in GSE83456 database. [file Image_2.tif]

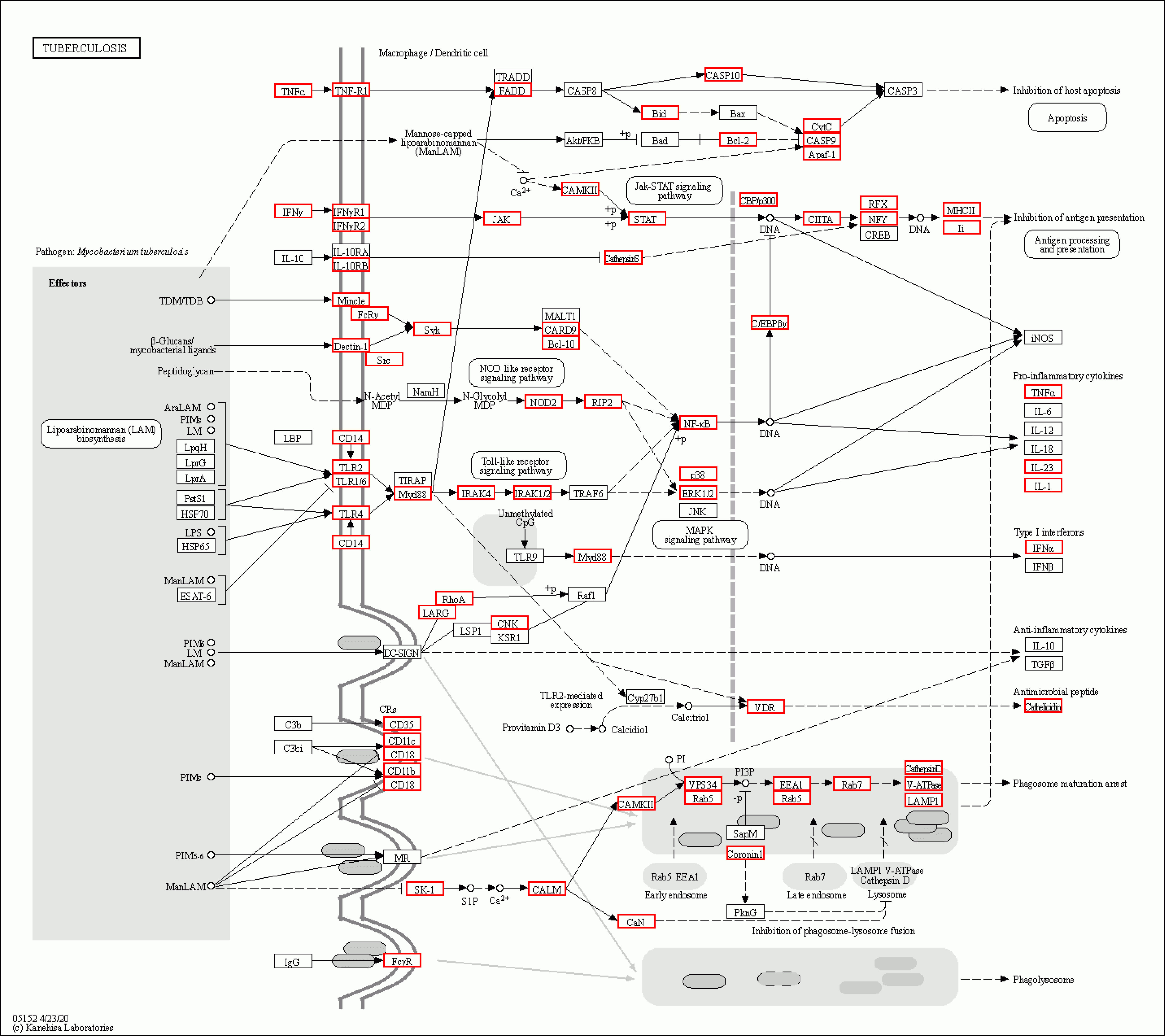

Supplement: Supplementary Figure 3 — Map of global signaling pathways associated with TB disease immunity. [file Image_3.tif]
